# Structural responses of metallic glasses under neutron irradiation

**DOI:** 10.1038/s41598-017-17099-2

**Published:** 2017-12-01

**Authors:** L. Yang, H. Y. Li, P. W. Wang, S. Y. Wu, G. Q. Guo, B. Liao, Q. L. Guo, X. Q. Fan, P. Huang, H. B. Lou, F. M. Guo, Q. S. Zeng, T. Sun, Y. Ren, L. Y. Chen

**Affiliations:** 1 College of Materials Science and Technology, Nanjing University of Aeronautics and Astronautics, Nanjing, 210016 P.R. China; 2 Department of Mechanical & Aerospace Engineering, Missouri University of Science & Technology, Rolla, MO 65409 USA; 3 Institute of Nuclear Physics and Chemistry, China Academy of Engineering Physics, Mianyang, 621900 P.R. China; 4 Center for High Pressure Science and Technology Advanced Research (HPSTAR), 1690 Cailun Road, Pudong, Shanghai, 201203 P.R. China; 5 Advanced Photon Source, Argonne National Laboratory, Lemont, Illinois 60439 USA

## Abstract

Seeking nuclear materials that possess a high resistance to particle irradiation damage is a long-standing issue. Permanent defects, induced by irradiation, are primary structural changes, the accumulation of which will lead to structural damage and performance degradation in crystalline materials served in nuclear plants. In this work, structural responses of neutron irradiation in metallic glasses (MGs) have been investigated by making a series of experimental measurements, coupled with simulations in ZrCu amorphous alloys. It is found that, compared with crystalline alloys, MGs have some specific structural responses to neutron irradiation. Although neutron irradiation can induce transient vacancy-like defects in MGs, they are fully annihilated after structural relaxation by rearrangement of free volumes. In addition, the rearrangement of free volumes depends strongly on constituent elements. In particular, the change in free volumes occurs around the Zr atoms, rather than the Cu centers. This implies that there is a feasible strategy for identifying glassy materials with high structural stability against neutron irradiation by tailoring the microstructures, the systems, or the compositions in alloys. This work will shed light on the development of materials with high irradiation resistance.

## Introduction

Designing nuclear materials that possess a high resistance to particle irradiation damage has been the subject of intense interest^1–5^. During service in fission reactors, the failures of nuclear materials are usually observed, in terms of swelling, hardening, amorphization, and embrittlement^6,7^. They are caused by the particle irradiation-induced changes of micro- to macroscopic-scale structures^8^. At the atomic scale, a primary knock-on atom is excitated by an incidental particle^9^. One primary knock-on atom can further excite a number of atoms in the bulk to move and collide with each other. This is the so-called effect of collision cascade (ECC)^10^. These excitated atoms become the interstitials and leave corresponding vacancies in the bulk, leading to the point defects. In principle, point defects are the universal primary structural damages in any condensed matter that undergoes particle irradiation. In crystalline materials, it is known that most of the irradiation-induced vacancies will be annihilated by the interstitials themselves, resulting in an annealing effect^11^. The residual vacancies in the bulk will migrate to the grain boundaries (GBs), and even to the interfaces, which are the effective sinks for defects^12,13^. Recently, it has been revealed that, in nanocrystalline copper, a more effective annealing of the vacancies by the interstitial atoms occurs. This is because the nano-scale GBs have a surprising “loading-unloading” effect^14,15^ that leads to enhanced irradiation resistance^16^.

As a new class of alloy materials, metallic glasses (MGs) have drawn intense interest because of their unique properties and amorphous structures^17–19^. Specifically, it is well known that there are no unit cells, grains, or GBs in glassy alloys, or even any well-defined defects in crystallography. Consequently, it is anticipated that such materials have relatively high resistance against particle irradiation. Since decades ago, a number of neutron, ion, and electron irradiation experiments were performed on MGs^20–23^. It was experimentally observed that the MGs demonstrated a high resistance to irradiation damage. For instance, unlike crystalline alloys, both the volume expansion (swelling) and the volume contraction, which have relatively low saturation values, were observed in the MGs^24^; and some irradiated amorphous alloys became even more ductile^20,25,26^, while irradiation-caused embrittlement in almost all of the crystalline alloys.

In some recent computational investigations using molecular dynamics (MD) simulation, when the irradiation response of MGs was studied from an atomic structural or mechanistic aspect^27^, it seems that amorphous structure is relatively stable under irradiation^28^. However, the mechanisms of particle irradiation (especially neutron) that damage the microstructure of MGs remain elusive. In a MG irradiated by neutrons, like interstitials and vacancies in crystals, there are some excitated atoms and corresponding vacant spaces left in MGs. We call them interstitial-like and vacancy-like defects^29^. Some open questions are thus raised: 1) will most of the irradiation-induced vacancy-like defects be annihilated by excitated atoms, just like vacancies annihilated by interstitials in (nano)crystalline alloys? 2) or will the vacancy-like defects distribute randomly in the bulk without any annihilation? 3) if annihilation of vacancy-like defects occurs, what is the evolution of microstructure in MGs? Addressing these issues will be helpful for determining the structural mechanism of the resistance against particle (such as neutron) irradiation damage in glassy alloys.

In this work, a feasible scheme for addressing these issues is developed, by performing a series of experiments, coupled with simulations, to investigate the microstructure of a neutron-irradiated Zr_2_Cu binary amorphous alloy. It is found that there are some unique structural responses of irradiation damages in this class of glassy materials.

## Results and Discussion

### Experimental data of synchrotron radiation-based measurements

#### Data of synchrotron radiation-based X-ray diffraction

**Figure 1:**
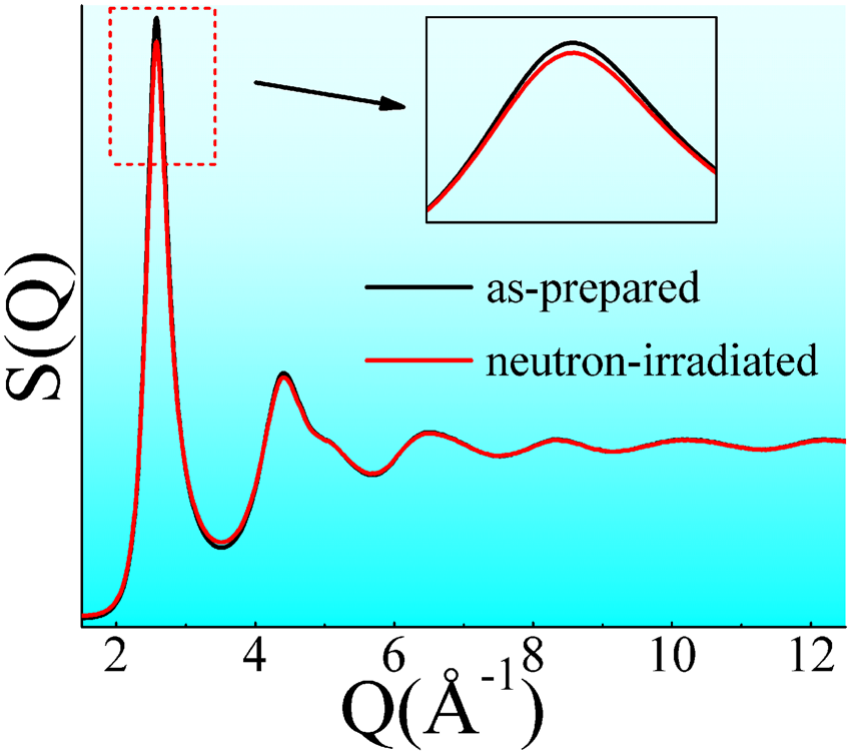
Synchrotron radiation XRD data of the as-prepared and the neutron-irradiated Zr_2_Cu samples. Here, structural factors, S(Q) curves, which were originated from the two-dimensional XRD patterns, are plotted. The insert is the highlight.

Both of the as-prepared and the neutron-irradiated Zr_2_Cu samples were measured by the synchrotron-radiation based high-energy X-ray diffraction (XRD) experiment. After normalization of the XRD data, the corresponding structural factors, (S(Q)s), were obtained and plotted in Fig. 1. The absence of any sharp peaks behind the first strong peak in the S(Q) curves adequately indicates that both of the structures of as-prepared and the neutron-irradiated Zr_2_Cu samples are full amorphous. In other words, neutron irradiation could not change the amorphous nature in Zr_2_Cu. In addition, we find that there are slight intensity differences in the first and the second peaks of S(Q)s of these two samples, implying that neutron irradiation caused slight or fine structural changes. To probe the direct structural change between these two samples from the diffraction data is expected. However, unlike crystal alloys, it is difficult to deduce direct or detailed structural information from the experimental diffraction signals by data normalization, index or direct fitting^30,31^. Therefore, further study is required.

#### Data of synchrotron radiation-based extended X-ray absorption fine structure

**Figure 2:**
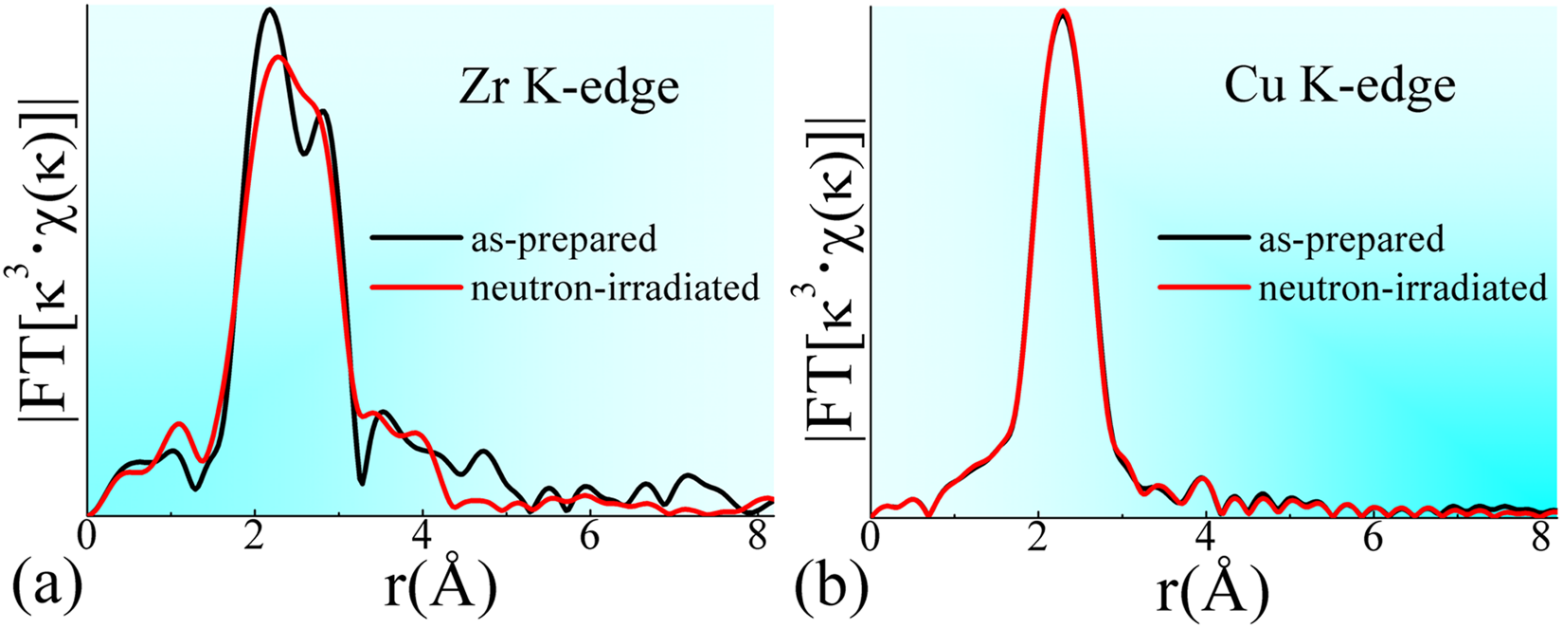
Normalizations of synchrotron radiation experimental data on the as-prepared and the neutron-irradiated Zr_2_Cu MGs, including: (**a**) Zr K-edge and (**b**) Cu K-edge partial radial distribution functions (PRDFs).

Partial radial distribution functions (PRDFs) of Zr and Cu K-edge, which were obtained from the extended X-ray absorption fine structure (EXAFS) experiments, are plotted in Fig. 2. It is revealed that there are detectable differences in the PRDFs of the Zr K-edge between these two samples. This indicates that some fine structural changes do appear after the ribbons are irradiated by neutrons, because an EXAFS measurement is an effective elemental-identified method for detecting a short-range order^32^. Unlike crystalline alloys, direct EXAFS fitting usually fails to detect short-range structural information in MGs, because their relatively complex short-range structures are tuned by topological atomic packing and chemical atomic interactions among heterogeneous atoms^30,32^.

In addition, we notice that there is a peak split in the first-shell Zr-edge partial PRDF of the as-prepared sample, while there is only one peak in that of the irradiated one, as shown in Fig. 2(a). Meanwhile, Zr-Zr and Zr-Cu bond lengths in the as-prepared sample are very similar to their counterparts of the irradiated one. It seems that there is a contradiction. Unlike crystalline alloys, the first-shell atomic distances in MGs are not discrete values, but average values. Neighbor atoms usually are randomly distributed around center atoms, exhibiting a Gaussian distribution. That is why there is one broad first-shell peak in many EXAFS PRDFs. But in some Zr-rich compositions such as Zr_2_Cu studied in this work, the length difference between Zr-Zr and Zr-Cu pairs probably contributes to a peak split in the first-shell of Zr-edge PRDF^33^. In a neutron-irradiated sample, the peak split in Zr-edge PRDF is weakened, probably due to the rearrangement of neighbor atoms around Zr centers, although the average Zr-Zr and Zr-Cu bond lengths are barely changed.

### Simulation upon synchrotron radiation data

Although direct normalizing, indexing or fitting the XRD or the EXAFS data fails to probe detailed structural information in MGs, simulating these synchrotron radiation experimental data is an effective approach for obtaining the reliable atomic structural models of MGs. In this work, a feasible scheme was developed that combined synchrotron radiation techniques with a series of simulations. This is similar to the previous work studying microstructure in MGs^34^. In detail, the normalized synchrotron radiation data, such as structural factor, S(Q), and both Zr and Cu K-edge EXAFS signals, were simulated simultaneously under the framework of Reverse Monte-Carlo (RMC)^35^. Subsequently, a MD simulation was performed to modify the structural model obtained via RMC, using a ZrCu chemical potential^36^ obtained from the embedded atom method (EAM)^37^, so that both computational (e.g., iterative fitting) and physical (e.g., chemical potential) considerations were included, ensuring the validity of this simulation.

**Figure 3:**
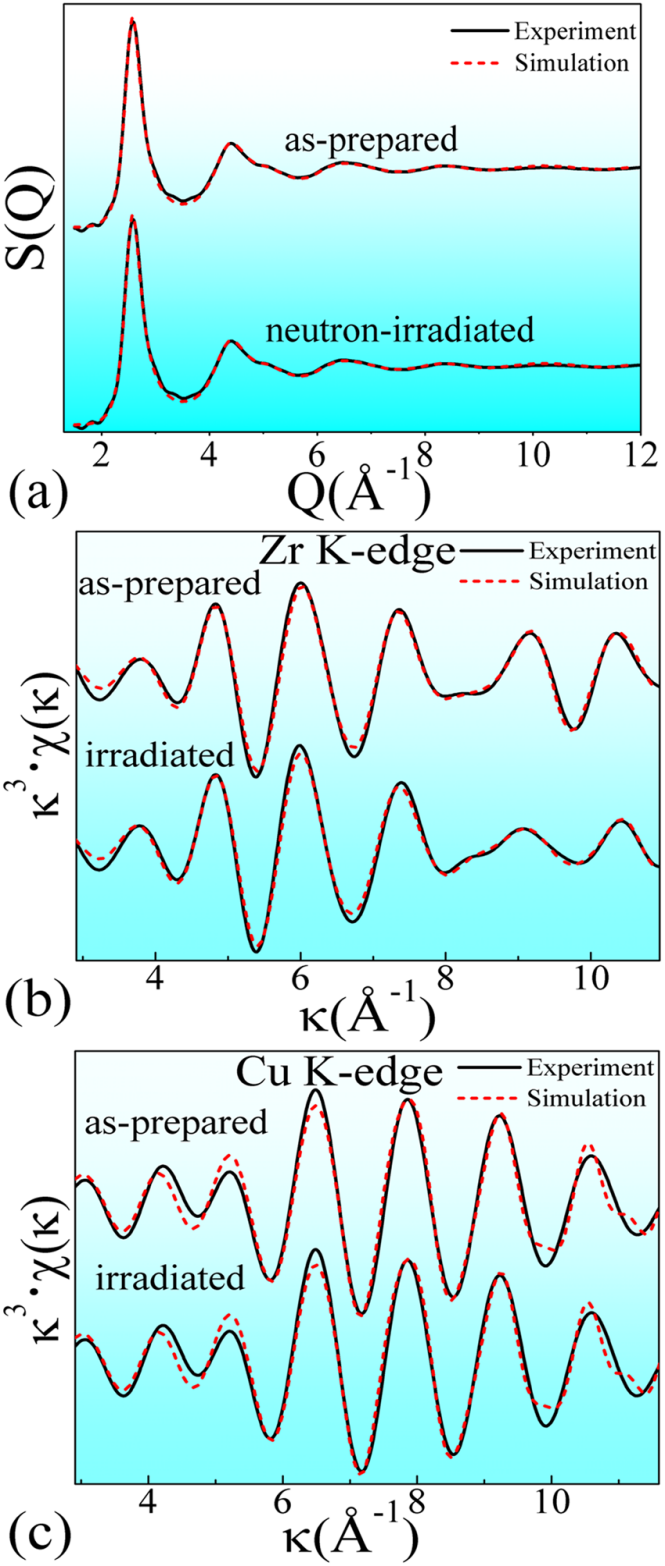
Comparison of the MD modified simulation and the synchrotron-radiation experiments for both of the as-prepared and the neutron-irradiated samples, including: (**a**) structural factor, S(Q), (**b**) Zr K-edge and (**c**) Cu K-edge EXAFS χ(κ) spectra. The solid and the dashed lines denote the experimental and the simulation data, respectively. The κ and the χ(κ) represent the photoelectron wave vector and the κ-space EXAFS signal, respectively.

**Figure 4:**
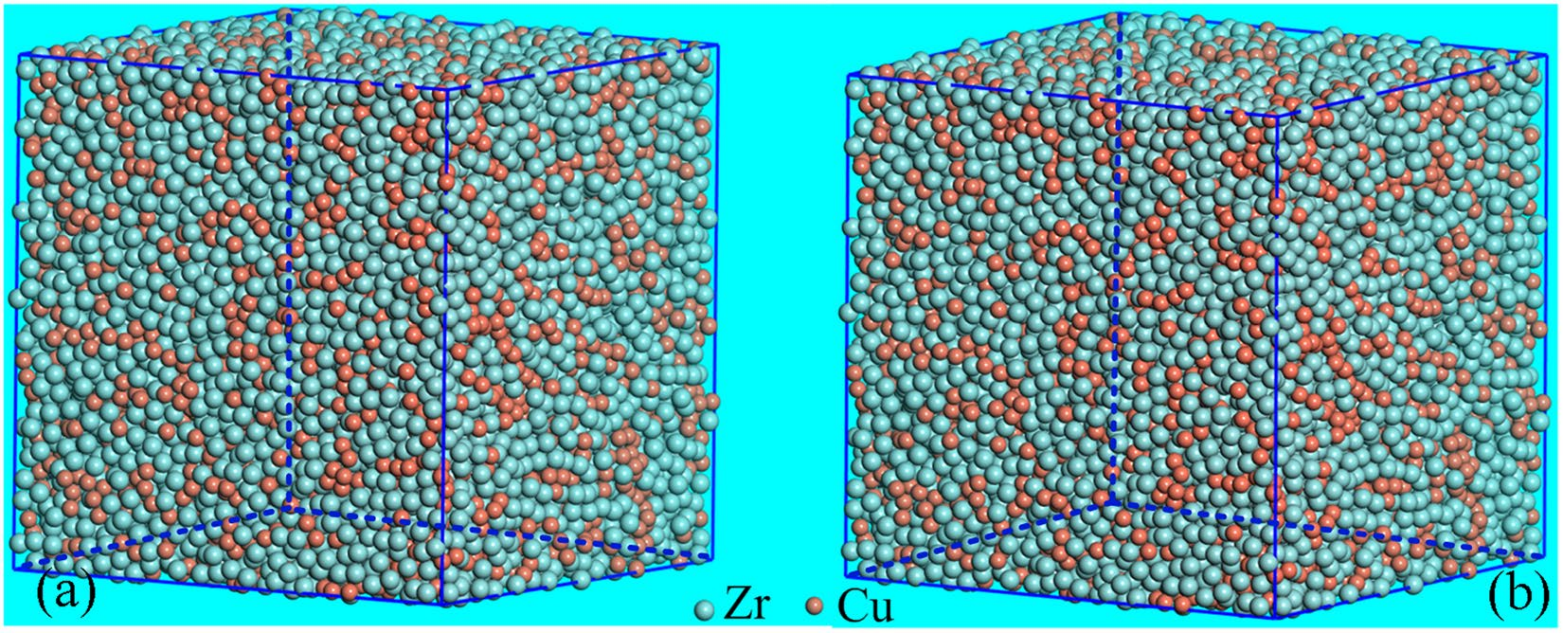
Three-dimensional configurations of structural models obtained by a MD modification of the RMC simulation, including: (**a**) as-prepared and (**b**) neutron-irradiated samples. The celadon and the red spheres denote the Zr and the Cu atoms, respectively.

Figure 3(a–c) show the experimental and the simulated curves of structural factor, (S(Q)), Cu and Zr K-edge EXAFS signals, respectively. Both of these simulated S(Q) and EXAFS curves fit well with the experimental ones, confirming that the MD simulation can successfully provide modified and reliable structural models. Figure 4 shows the simulated reliable structural models that resemble the real microstructure of MGs. Because all the atoms are “frozen” in these simulated structural models, the positions and the sizes of atoms can thus be determined, making it possible to probe detailed structural information.

### Short-range structural information

#### Atomic-level structural information

From the simulated structural models, atomic structural information including the coordinate numbers (CNs) and the atomic pair distances were calculated and listed in Table 1. For the as-prepared ZrCu sample, Zr-Zr, Zr-Cu, and Cu-Cu pairs have bond lengths of 3.21, 2.96, and 2.61 Å, respectively. The CN values around Zr and Cu centers are 13.8 and 11.3 respectively. According to Miracle’ efficient cluster-packing model^38^, the optimal CNs around Zr and Cu in Zr_2_Cu are estimated to be 14.1 and 11.0, respectively, which are similar to the present values. Moreover, it is worth noting that there is no detectable difference in the CNs or atomic pair distances between the as-prepared and the neutron-irradiated ZrCu samples.

**Table 1:** The atomic structural information for the as-prepared and the neutron-irradiated ZrCu samples, including: first-shell atomic distances (R) and CNs around Zr and Cu atoms, respectively. They were obtained from the MD modified structural models.

Samples	R (±0.01 Å)			CNs (±0.1)					
	Zr-Zr	Zr-Cu	Cu-Cu	Zr-Zr	Zr-Cu	Zr_total_	Cu-Zr	Cu-Cu	Cu_total_
As-prepared	3.21	2.96	2.61	8.2	5.6	13.8	6.8	4.5	11.3
Irradiated	3.20	2.96	2.61	8.2	5.6	13.8	6.8	4.5	11.3

#### Cluster-level structural information

**Figure 5:**
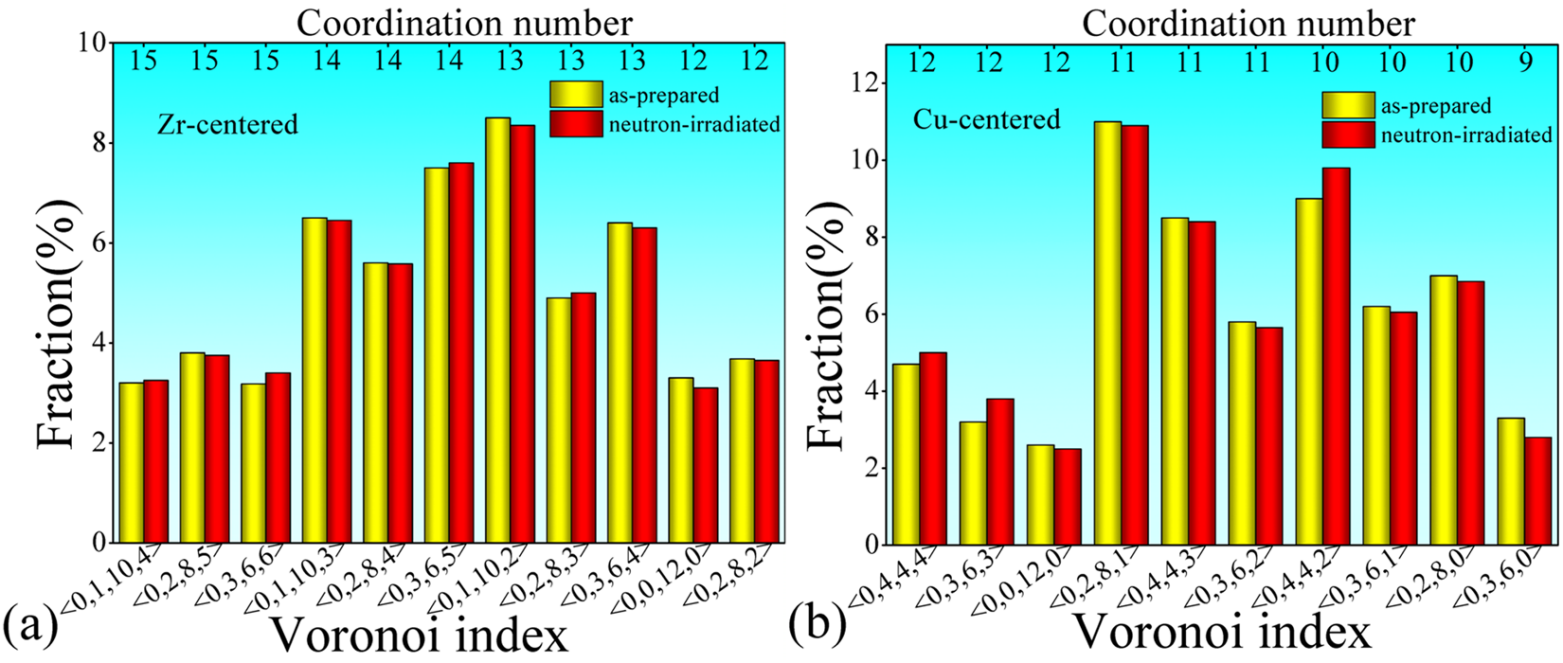
Distribution of the major VCs centered with (**a**) Zr and (b) Cu atoms. Only those whose fractions are larger than 2% are selected. The CN value denotes the number of the shell atoms of VCs.

Cluster with one center atom and some shell atoms was regarded as a standard structural unit which can indicate the short-range order in MGs^30,32^. In this work, Voronoi tessellation method was applied for extracting Voronoi clusters (VCs) in these simulated MG models^33,39^. The distributions of major Zr- or Cu-centered VCs whose fractions are larger than 2% are plotted in Fig. 5. It is found that the CNs around Cu and Zr centers are ranged from 9 to 12 and 12 to 15, respectively. By comparing the fraction of VCs, it is found that Cu and Zr atoms in these five samples are likely to be centered in < 0,2,8,1 > , < 0,4,4,2 > , < 0,4,4,3 > and < 0,1,10,2 > , < 0,3,6,5 > , < 0,1,10,3 > , < 0,3,6,4 > clusters, respectively. Since < 0,1,10,2 > is a typical icosahedral-like cluster^39,40^, it indicates that icosahedral-like clusters are apt to be formed around Zr (the solute) rather than Cu (the solvent) in this Zr-rich composition. Concerning the ideal icosahedron, < 0,0,12,0 > , its fraction is sensitive to the cut-off value of atomic distances in VCs, and is easy to transform into other VCs such as < 0,2,8,1 > or < 0,1,10,2 > , by removing or adding one neighbor atom. The fraction of < 0,0,12,0 > in this work is even smaller than that of previous work^41^, is probably due to the different cut-off values adopted. The detected < 0,0,12,0 > VCs in both this work and previous work^41^ have a rather low fraction less than 5%.

In addition, it is worth noting that there also is no detectable difference in the distributions of major VCs between the as-prepared and the neutron-irradiated ZrCu samples. In other words, the detectable differences in Zr-edge PRDFs are not attributed to any change of these cluster structural parameters. Since neither atom-level nor cluster-level short-range structures directly response to the neutron irradiation in MGs, other structural features or parameters which can response to the neutron irradiation should be studied. For instance, we wonder whether or not vacancy-like defects induced by neutron irradiation are preserved in the ZrCu sample.

### Free volumes detected via positron annihilation spectroscopy

In order to detect the possible vacancy-like defects induced by neutron irradiation, a positron annihilation spectroscopy (PAS) measurement was performed. The deduced positron lifetime values for both samples are listed in Table 2. In previous work studying the PAS data in ZrCu-based MGs, the positron lifetime values for Zr and Cu pure metals usually were used for comparison^42,43^. Therefore, the positron lifetime values for Zr and Cu pure metals also are listed in Table 2. We notice that one short lifetime, with a small value about of 0.14 ns in the as-prepared ribbons, has a rather high intensity value of 86%. This is consistent with the experimental observations in previous work studying MGs^43–45^. Such a short lifetime indicates the excess free volumes^46^, and is obviously shorter than that denoting the monovacancies^43,47^. It is worth noting that, in the irradiated sample, there is only one short lifetime with a value of about 0.15 ns, which is obviously shorter than those corresponding to the monovacancies in Cu (0.18 ns) and Zr (0.25 ns) metals^43,47^. This is interesting because it implies that no vacancy-like defects caused by ECC could be preserved after the structural relaxation in MGs, while it is known that there should be some residual monovacancies in (nano)crystalline alloys. In previous work, it has been theoretically predicted that irradiation-induced vacancy-like defects are unstable in a Lennard-Jones amorphous solid^48^. In addition, this relatively short life time increases from 0.14 to 0.15 ns, implying that the expansion of free volumes occurs in an irradiated sample. In both of the as-prepared and the irradiated samples, a relatively long lifetime of 0.32 ns is observed, which should relate to some intrinsic microvoids or multivacancies^49^ whose sizes are much larger than the vacancy-like defects. Moreover, a long life time of 1.9 ns is found in both samples. This is caused by the positrons trapped in some caves of the ribbon surfaces, apertures, and so on. The lifetime of 0.32 ns, or 1.9 ns, has almost the same intensity in these two samples, indicating that neutron irradiation in this work does not affect microvoids or caves in MGs.

**Table 2:** Positron lifetimes of as-prepared and neutron-irradiated Zr_2_Cu glassy alloys and other experimental values of pure metals provided elsewhere. τ and I are the lifetime and its intensity, respectively.

Samples	τ_1_(ps)	τ_2_(ps)	τ_3_(ps)	I_1_(%)	I_2_(%)	I_3_(%)
As-prepared	140	318	1943	86.0	12.8	1.2
Irradiated	151	320	1946	85.8	13.0	1.2
ZrCuNiAlNb	152[49]	360[49]				
Cu	180[47]					
Zr	252[45]					

### Annihilation vacancy-like defects by free volumes

#### Detecting void spaces among atoms

**Figure 6:**
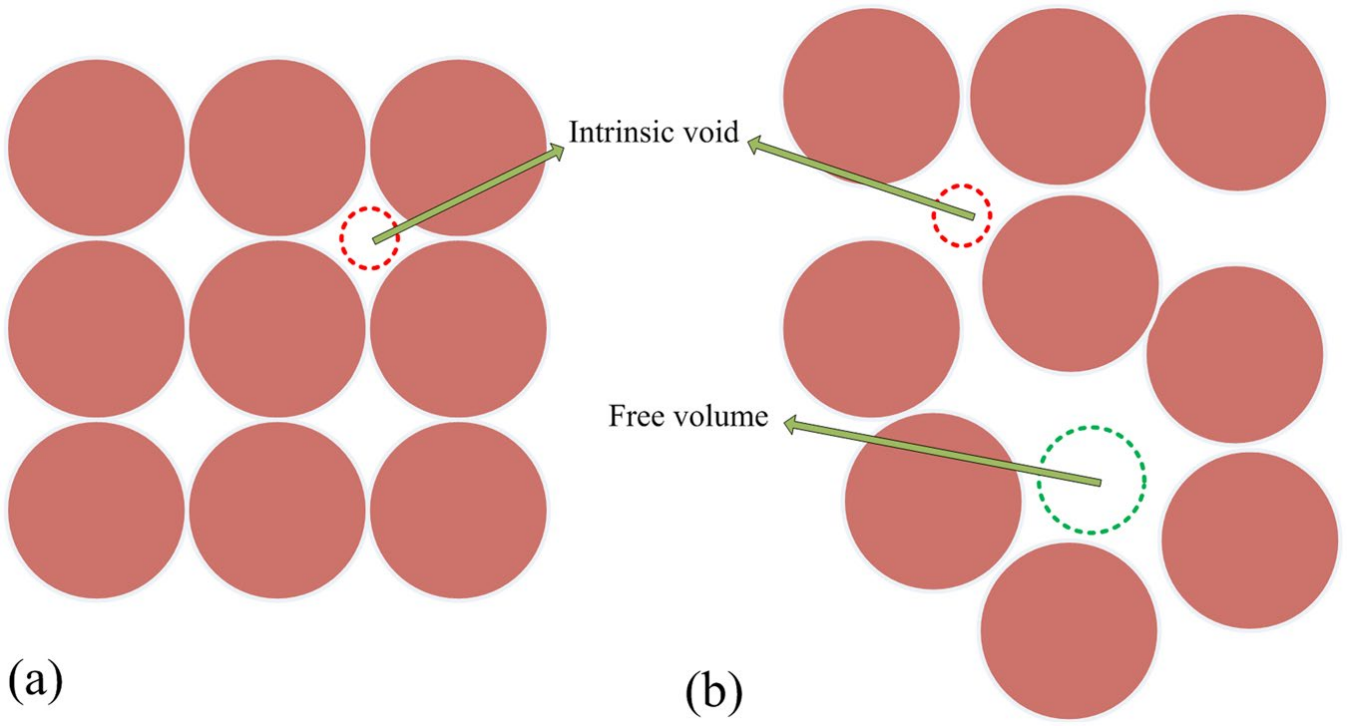
An illustration roughly indicating the intrinsic voids in (**a**) a crystal, and the intrinsic voids coupled with free volumes in (**b**) a MG. Here we stress that the sphere marked with “Free volume” does not indicate the size of a free volume, but its site.

In a crystalline alloy, atoms usually are regarded as hard spheres, are bonded and packed densely in space, leaving a number of void spaces among them. In other words, voids spaces are those which are not spatially occupied by atoms in the microstructure of an alloy. These voids spaces are called as intrinsic voids. In an amorphous alloy, voids spaces are made up of both intrinsic voids and free volumes^50^. Figure 6 is an illustration roughly indicating the intrinsic voids in a crystal and the intrinsic voids coupled with the free volumes in a MG.

A computational method for detecting void spaces in both of the as-prepared and the neutron-irradiated samples was developed here. A hollow sphere for probing all of the possible void spaces among neighboring atoms in simulated structural models was designed. This sphere has a changeable radius and is allowed to move randomly in a structural model until it touches any of the position-determined atoms. Although intrinsic voids occur in both crystalline alloy and amorphous alloy, and are involved in the detected void spaces, this computational method can provide free volume information. To illustrate this, the intrinsic voids in a structural model of the corresponding Zr_2_Cu crystalline phase were calculated, as shown in Table 3. It is worth noting that there is no void with a radius lager than 0.4 Å. It is known that intrinsic voids are those which are intrinsically present in an atomic-dense packing structure for both crystalline and glassy alloys, and the sum of the intrinsic voids and the free volumes is the total void space in a MG^50^. Therefore, it suggests that any hollow sphere with a radius larger than 0.4 Å can denote free volume. All of the voids whose radii are larger than 0.4 Å were sorted, and their distributions are shown in Table 4. It is interesting that there are more voids with a size larger than 0.4 Å in the neutron-irradiated sample, implying that the quantity or the average size of free volumes increases after neutron irradiation. This is consistent with the PAS result.

**Table 3:** Size (the radius) distribution of intrinsic voids detected from a structural model containing more than 50,000 atoms of the Zr_2_Cu tetragonal phase.

Size (Å)	Number of voids	Percentage
<0.2	0	0%
0.2~0.3	47040	66.7%
0.3~0.4	23520	33.3%
>0.4	0	0%

**Table 4:** Distributions of all detected void spaces whose radii were larger than 0.4 Å in both of the as-prepared and the neutron-irradiated Zr_2_Cu samples. These void spaces were not probed in the corresponding Zr_2_Cu crystalline phase, so they could indicate the free volumes in MGs.

Samples	The number of the voids with different radii (Å)					
	0.4~0.5	0.5~0.6	0.6~0.7	0.7~0.8	0.8~0.9	>0.9
As-prepared	9386	3197	702	74	9	0
Irradiated	9432	3240	733	127	14	0

#### Self-healing by free volumes

**Figure 7:**
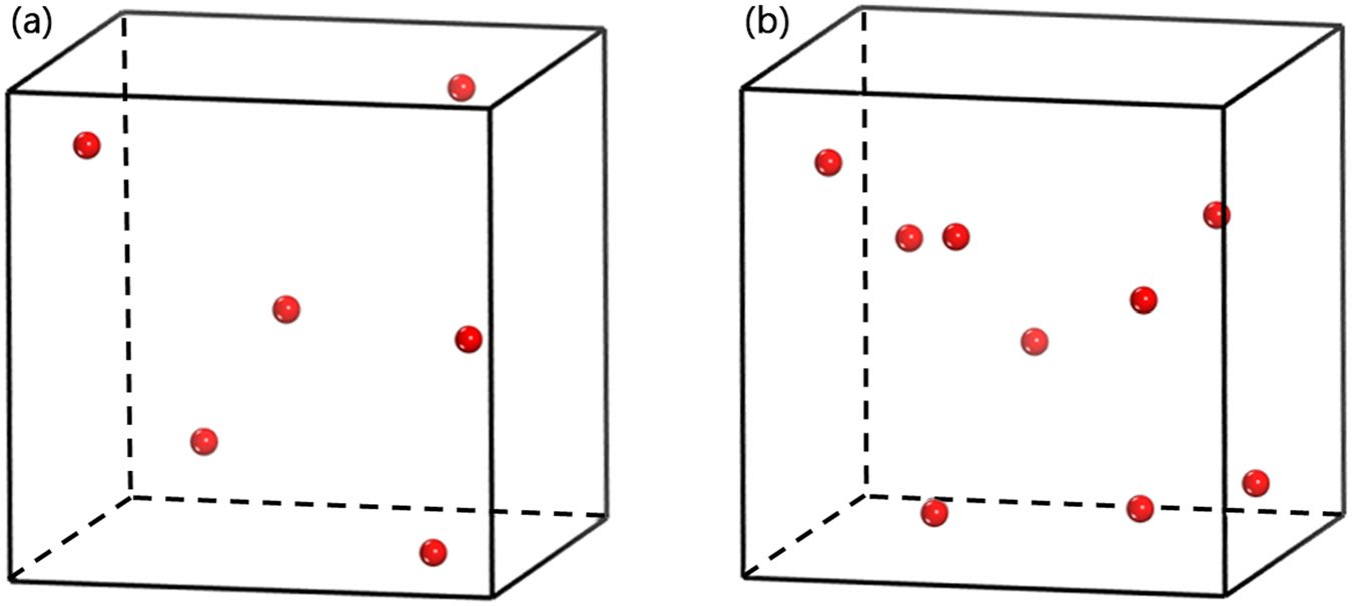
Distributions of the large voids whose radii ranged from 0.82 to 0.90 Å in the MD modified structural models for (**a**) the as-prepared and (**b**) the neutron-irradiated Zr_2_Cu samples. The red spheres denote the large voids with different positions between the as-prepared and the neutron-irradiated Zr_2_Cu MGs, These large voids are not intrinsic voids, but free volumes, because it is confirmed that the radii of the intrinsic voids in the corresponding Zr_2_Cu crystalline phase are not larger than 0.4 Å.

We notice that there is no detectable hollow sphere when the probing radius is larger than 0.9 Å. The small atom (Cu) in this binary system has a Goldschmidt radius of 1.28 Å, which is much larger than 0.9 Å. Therefore, we can conclude that there is no vacancy-like defect in both samples. This indicates that rearranging free volume, rather than preserving vacancy-like defects, is an interesting structural response in glassy alloy after neutron irradiation. To illustrate this, the structural models containing relatively large free volumes (a great number of relatively small ones are not shown here) are depicted in Fig. 7. It is found that, compared with the as-prepared sample, both the number and the positions of these large free volumes do change in the neutron-irradiated one. The average size of free volumes also is calculated, and its value in the as-prepared sample is 0.487 Å, slightly larger than that (0.478 Å) in neutron-irradiated MG.

**Figure 8:**
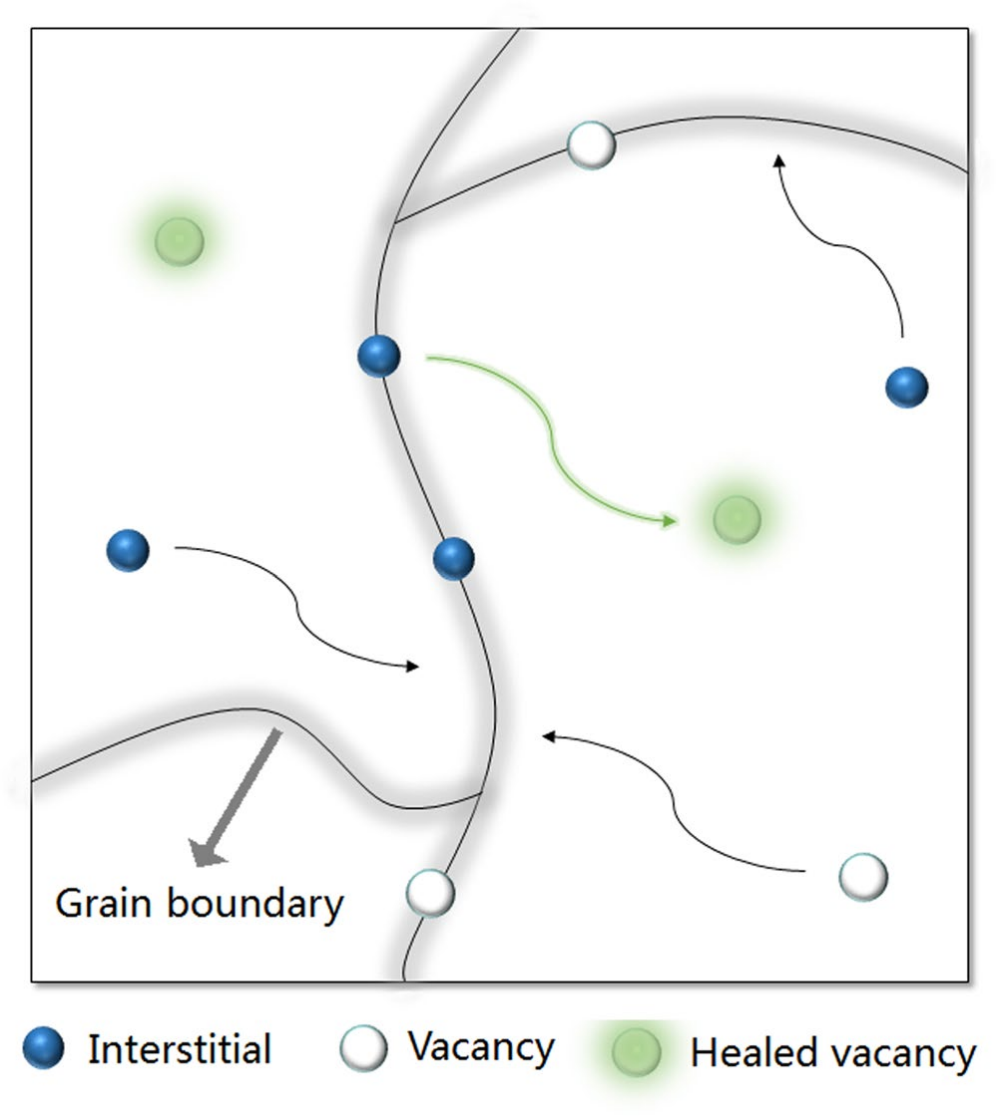
An illustration indicating how irradiation-induced vacancies appear, and are recovered or absorbed in (nano)crystalline alloys.

In principle, vacancies or vacancy-like defects caused by ECC should occur in any condensed matter, until they are annihilated^9,13,15^. In this work, by performing both PAS experiment and RMC/MD simulations, we found no vacancy-like defect in the neutron-irradiated sample, indicating that monovacancies are completely annihilated after structural relaxation. This full defect self-healing phenomenon should be explained. In (nano)crystalline alloys, it is revealed that vacancies are recovered by interstitials during the diffusion of vacancies towards the GBs, or interstitials are emitted from the GBs to heal the vacancies in the bulk^13,15^. This is the atomic-scale annealing mechanism of the irradiation damage in (nano)crystalline alloys. Figure 8 shows an illustration indicating how irradiation-induced vacancies appear, migrate, and are recovered or absorbed in (nano)crystalline alloys. Concerning amorphous alloys, there are no GBs which are the absorbing and emitting sources of interstitials in crystals^18^. Therefore, where the excitated atoms are trapped or emitted is a mystery, let alone revealing the healing mechanism of vacancy-like defects. We have revealed that free volumes will rearrange for healing the vacancies during structural relaxation. Such free volume rearrangement probably relates to the movement of atoms in the bulk, in particular those surrounding a vacancy-like defect to form one vacancy-centered cluster^15^. Therefore, studying the change of vacancy-centered clusters is required, which may be helpful in clarifying the underlying annealing mechanism.

**Figure 9:**
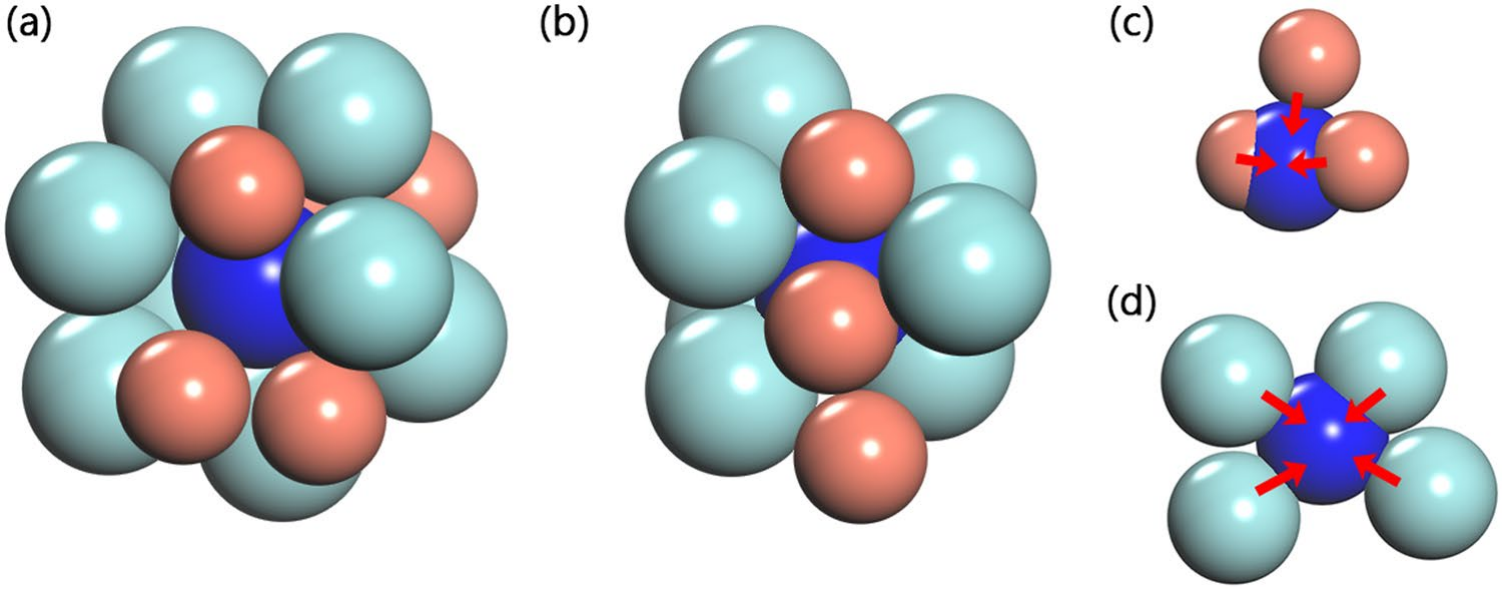
Configurations of the cluster containing an “artificial” vacancy-like defect in (**a**) the as-prepared model and (**b**) the further MD simulated model. Atoms overlapping with this vacancy-like defect in the MD simulated model are shown in (**c**) and (**d**). To clearly show all of the atoms overlapping with this vacancy-like defect, they are separately shown in (**c**) and (**d**). The number on each atom denotes the serial number, and V denotes the vacancy-like defect. The vacancy-like defect, Zr, and Cu atoms are shown in blue, celadon, and red colors, respectively.

**Figure 10:**
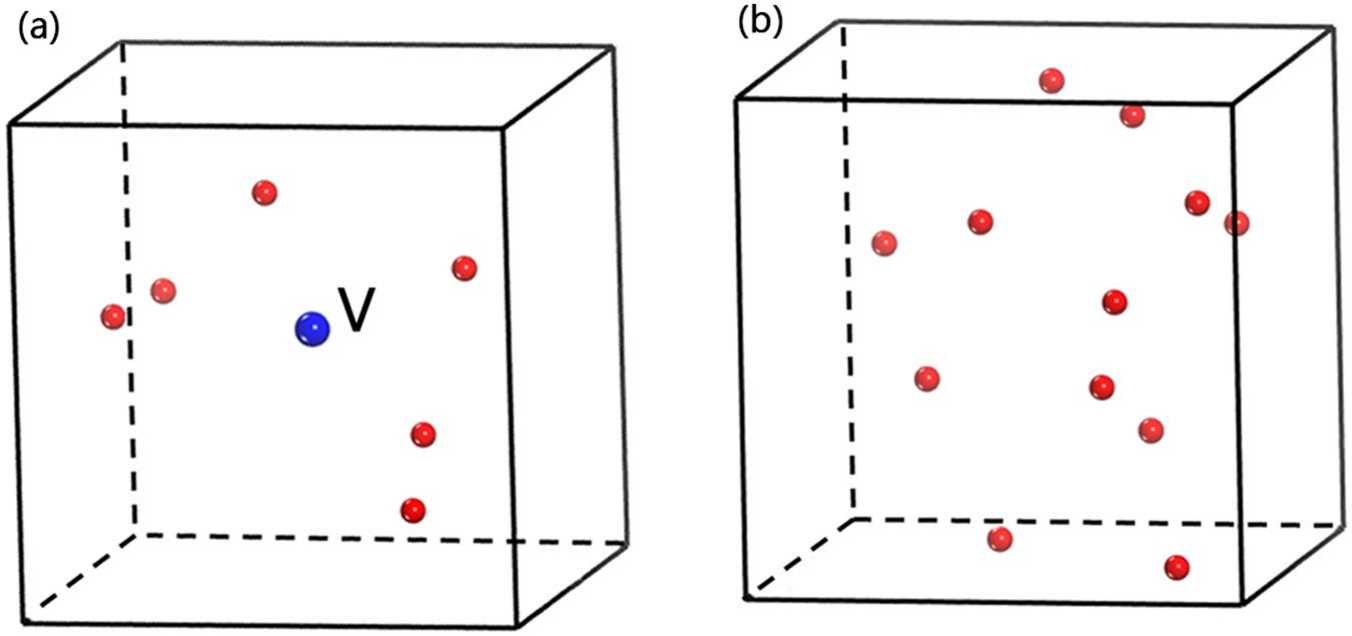
Distributions of the large free volumes whose radii are larger than 0.80 Å in the structural model containing an “artificial” monovacancy (**a**) before the MD simulation and (**b**) after the MD simulation. The red spheres denote the large free volumes with different positions between the as-prepared and the neutron-irradiated Zr_2_Cu MGs. The blue ball, marked with V, is the “artificial” vacancy-like defect.

Another MD simulation was applied to study the evolution of a vacancy-centered cluster. Based on the simulated structural model of the as-prepared sample, a vacancy-centered cluster was created by removing a center atom, as shown in Fig. 9. This unstable structural model, containing this vacancy-centered cluster, will get stable after structural relaxation under the MD simulation, so that the change of this vacancy-like defect could be probed. It is found that there is no vacancy-like defect in this further simulated model. This indicates that this created vacancy-like defect collapses or disappears. By comparing the snapshots before/after the MD simulation, we could see that there is an obvious change of the created vacancy-centered cluster. The space where the vacancy-like defect is located is compressed by several neighboring atoms, but not occupied completely by one atom. In other words, there is a concurrent compression mechanism to annihilate the vacancy-like defect by its neighboring atoms. Since the irradiation-induced vacancy-like defect is annihilated by neighbor atoms which can move closer to the center of the vacancies, it is apparent that the free volumes in the structural model will rearrange and get larger on the average. The average size of free volume in the structural model before/after the MD simulation was calculated. It is found that their sizes do increase after the MD simulation, implying the expansion of free volumes. To further illustrate the rearrangement of free volumes, the distributions of free volumes with relatively large sizes in both structural models are plotted in Fig. 10. It is shown that the number and positions of these large voids have changed, in accordance with both of the PAS measurements and the simulation results. In addition, as shown in Fig. 10(b), the disappearance of the created vacancy-like defect in the structural model after the MD simulation clearly indicates a correlation between the annihilation of a vacancy-like defect and the rearrangement of free volumes.

Here we emphasize that although we only calculated the structural change around one vacancy-like defect, while ECC usually causes a few of vacancy-like defects simultaneously, this simulation can illustrate how a vacancy-like defect is annihilated by rearrangement of free volumes.

Based on the results mentioned above, we can discuss the full self-healing in MGs. In a crystal, the irradiation-induced vacancies are not stable in both structural and energetic perspectives, so that vacancies move from the bulk towards the GB absorber along the crystal lattice^13,15^, unless that they are recovered by interstitial atoms. In a nanocrystal, although the relatively small particles contribute to a greatly enhanced self-healing efficiency of point defects, some unhealed vacancies will keep on moving until they are absorbed at the GBs, leading to some permanent point defects. In an amorphous alloy, the neutron irradiation-induced vacancy-like defects also cause problems of structural instability and energy imbalance that are resolved when these vacancy-like defects are recovered or absorbed. Unlike vacancies that migrate from the bulk to the GBs (sinks) in a crystal, the vacancy-like defects in an amorphous structure have no specific destination to move toward, since there is no crystal lattice or unit cell, or in particular no GB to sink^18^. Therefore, there is probably no priority for any of the neighbor atoms to enter the vacancy-like defect alone and leave a new vacancy-like defect nearby, like the migration of a vacancy in crystals. In other words, a vacancy-like defect does not move with the aid of any single atom, but is an arrest to make some of the neighbor atoms move concurrently closer to it and crush it, until there is a new balance of structure and energy. As a result, this vacancy-like defect disappears rather than moves on, resulting in a full healing.

### Elemental-specific free volume rearrangement

In addition, as shown in Fig. 2, there are detectable differences in the PRDFs for the Zr K-edge between these two samples, although there is almost no difference for the Cu K-edge. This indicates that neutron irradiation have led to changes in local structures around Zr centers, rather than Cu atoms. This is quite surprising, because it is not expected that there would be a large difference in the probability of exciting Cu and Zr atoms to move by ECC, because there is not a large difference in the threshold displacement energy of Zr and Cu atoms^51^. As listed in Table 1, no difference is found in the coordinate numbers or atomic pair distances of the as-prepared and the neutron-irradiated ZrCu samples. In other words, the detectable difference around the Zr atoms is not attributed to any change of these atomic structural parameters. On the other hand, although some atomic structural features such as CNs are not sensitive to the irradiation, we have revealed that the arrangement of free volume is a structural response of the neutron irradiation. Therefore, it is supposed that the changes/invariabilities of local structures around the Zr/Cu centers are caused by the rearrangement/invariability of the free volumes around the Zr/Cu centers.

To validate this scenario, the change of the free volumes around Zr or Cu centers, was calculated and listed in Table 5. Because EXAFS PRDFs of MGs usually have a strong peak that denotes the first-shell atomic distances whose cut-off values range from about 3.3 to 3.7 Å, a Zr-, or a Cu-centered sphere with a radius of 3.5 Å was adopted. In this sphere, the total volume of free volumes was calculated. It is worth noting that, after neutron irradiation, the total free volumes with this sphere increase of 5.8% for Zr centers, while the counterpart of Cu centers only increases about 1.5%. This explains why there is a relatively large/small difference in the first-shell distribution of the Zr/Cu K-edge EXAFS PRDFs between the as-prepared and the neutron-irradiated samples. Changes in both of the free volume calculation and the EXAFS signals confirm that the arrangement of free volumes mainly occurs around Zr centers, rather than Cu atoms. In other words, the arrangement of free volumes is an element-specific structural response of neutron irradiation damage. Since we have revealed that the arrangement of free volumes is an intrinsic irradiation-induced structural behavior in MGs, it is feasible to enhance the resistance against irradiation in glassy materials by designing alloy systems or compositions with constituent elements that are less sensitive to the arrangement of free volumes.

**Table 5:** Information about the free volumes around Zr or Cu centers in the as-prepared and the neutron-irradiated samples. All of the free volumes whose distances to the Zr or Cu centers were smaller than 3.5 Å were selected. V_a_, N, V_ra_, and V_as_ denotes the average volumes and the number of free volumes, the total volumes of free volumes in the as-prepared and the neutron-irradiated samples, respectively.

Center	Samples	V_a_ (±0.001Å^3^)	N (±0.01)	V_a_*N	(V_ra_ − V_as_)/ V_as_
Zr	As-prepared	0.466	16.43	7.703	5.80%
	irradiated	0. 478	17.05	8.150	
Cu	As-prepared	0.455	16.11	7.330	1.50%
	irradiated	0.459	16.21	7.440	

### Potential studies of free volumes in MGs under irradiation

Free volume is a specific structural parameter in amorphous or complex materials. It has been found that free volumes do change in some other amorphous or complex materials, such as silica glasses^52,53^, polymers^54,55^, colloids^56^, and so on, when these materials suffer from irradiation of ions, electrons, or neutrons. Therefore, the present work probably can be extended to a number of amorphous or complex materials possessing free volumes. In addition, unlike crystalline alloys, MGs have some similar structural features of the above-mentioned materials, such as no long-range order. On the other hand, like crystalline alloys, MGs have dense atomic packing structures and possess excellent mechanical and thermal properties, due to inherent metallic bonding. Therefore, it is reasonable that this special class of alloys have some unique structural responses to irradiation damage. Furthermore, since it has been proposed that free volume is a key factor that affects glass formation and mechanical properties in alloys^57–59^, these unique structural responses to irradiation (changes in free volumes in particular) are expected to explain both structural and mechanical changes in MGs.

### Potential use of MGs in irradiating environment

Although we have revealed that, compared with crystalline alloys, MGs have a more effective self-healing mechanism that can fully annihilate the irradiation-induced vacancy-like defects, we should carefully assess the potential risks of MGs for application in irradiating environment. In a real nuclear fission factor, besides irradiation damage, nuclear materials have to survive under extreme conditions, including high temperature, high stress, corrosion, aging, and so on^1–3^. Both crystalline alloys and MGs have their advantages as well as disadvantages, that they probably will be damaged more or less after a long-term service in such complex and critical environment. How to design modified materials to survive long enough in critical environment should be a key issue to be addressed in the future. For instance, designing complex materials based on traditional crystalline alloys and the amorphous alloys probably is a choice.

## Conclusions

In summary, the microstructural changes of MGs induced by neutron irradiation were investigated in this work using a series of experiments, simulations, and calculations. There are some interesting structural responses. First, unlike crystalline alloys, there is a full structural self-healing effect in MGs in that the irradiation-induced vacancy-like defects are completely annihilated, leading to rearrangement of free volumes. Second, the rearrangement of free volumes is an element-specific structural response, which mainly occurs around Zr atoms in the ZrCu binary alloy. This work not only reveals the underlying mechanisms of the structural change induced by neutron irradiation, but it also provides an in-depth understanding essential for evaluating and selecting alloy materials with high resistance to irradiation.

### Experimental and simulation methods

#### Composition selection

The ZrCu alloy system was selected as a research prototype in the present work, for the following reasons: 1) Zr-based crystalline alloys (such as Zr-2 and Zr-4) have been widely applied as nuclear materials^60^, and they have suffered from neutron-irradiation damage during service. Studying the resistance of irradiation damage in the corresponding Zr-based amorphous alloys is desired; 2) ZrCu is a typical binary alloy system available for detecting the microstructure^30^, with a broad composition region (30–80 at.% for Zr) forming MGs^61^. In this work, although Cu-rich composition such as Cu_64_Zr_36_ has better glass-forming ability, a Zr-rich composition, Zr_2_Cu, was selected, because Zr-based alloys rather than Cu-based ones are typical nuclear materials.

#### Sample preparation

A Zr_2_Cu alloy ingot was prepared by arc melting the mixture of Zr [99.95 wt.%] and Cu [99.98 wt.%] elements in Ti-gettered high-purity argon atmosphere^40^. The ingot was melted at least five times in order to ensure compositional homogeneity. The corresponding amorphous ribbons were fabricated by melt-spinning, producing a cross section of 0.04 × 2 mm^2^. In this work, amorphous ribbons rather than rods were studied, because: 1) based on ribbons samples we could perform synchrotron radiation experiments^30^; 2) amorphous ribbons rather than amorphous rods could be fabricated in the Zr_2_Cu composition.

#### Neutron irradiation experiment

A fast neutron irradiation experiment was performed at the Chinese Fast Burst Reactor-II (CFBR-II), the fuel of which was highly enriched uranium. The highest temperature of the core of the reactor was only about 50°C for both modes. Some of the as-prepared Zr_2_Cu ribbons were enveloped by a sheet of tissue paper and stuck tightly to the support plate of the CFBR-II reactor. During the irradiation, the CFBR-II reactor was operated on a steady state power mode or a pulsed power mode. The temperature increase of these ribbon samples was not more than 10 degrees centigrade. The ribbons were irradiated with an accumulative neutron fluence of up to 1.0 × 10^15^ n/cm^2^, and the fast neutrons had a relatively high average kinetic energy of 1.12 MeV, so that it was possible to detect irradiation-induced structural change at the atomic scale.

#### Positron annihilation measurement

PAS is an useful experimental tool for probing vacancies, voids, and cavities in various materials, including the amorphous alloys^45,62^. In this work, the PAS measurement was performed using a conventional fast-slow lifetime spectrometer with a time resolution of 225 ps (FWHM) at room temperature. The spectrum was accumulated over 1.5 × 10^6^ coincidence counts and analyzed by using the LT v.9.0 program^63^. A water solution of ^22^NaCl (an activity of 925 kBq) was evaporated onto the Kapton foil, so that a positron source could be obtained. The positron source was tightly sandwiched between two identical samples, which were the ribbons piled up to obtain an overall dimension of 10 mm × 10 mm × 0.25 mm, so that all positrons could be captured. After a standard data-reduced procedure, the positron lifetime spectra for both samples were analyzed by a three component analysis^49^.

#### Synchrotron radiation-based experiments

Room temperature XRD measurements were performed for both samples, using a high-energy synchrotron radiation monochromatic beam (about 100 keV) on beam line 11–1D-C, of the Advanced Photon Source in USA. Furthermore, using a transmission mode, Zr and Cu K-edge extended X-ray absorption fine structure spectra were measured at the beam lines, BL14W1, in the Shanghai Synchrotron Radiation Facility of China, and, U7C, in the National Synchrotron Radiation Laboratory of China. Each EXAFS measurement was repeated at least five times to exclude any occasionality. The EXAFS spectra were normalized via a standard data-reduced procedure, employing the software Visual Processing in EXAFS Researches^64^.

#### Reverse Monte-Carlo simulation

Synchrotron radiation-based Zr and Cu K-edge EXAFS data were simulated in RMC, using the software RMCA^30^. The initial cubic boxes were built containing more than 50,000 randomly distributed Zr and Cu atoms, according to the Zr_2_Cu composition. During one RMC simulation, the atoms moved randomly within a determined time interval. The experimental data were compared to the simulation with an iterative calculation^65^. Once the simulation and experimental data converged, the simulation was stopped, and all of the atoms were “frozen” in the cubic box. The result was an atomic structural model available for further analysis.

#### Molecular Dynamics simulation

Based on the RMC simulated structural models of both of the as-prepared and the neutron-irradiated samples, a MD simulation was applied to obtain modified models, by using the program of large-scale atomic/molecular massively parallel simulator (LAMMPS)^66^. These two models contain more than 50.000 atoms, matching the Zr_2_Cu composition. Each model is a cube with side length of 14.1 nm. The force field adopted in this simulation was the EAM^37^, using a ZrCu potential suitable for simulating amorphous structure^36^. The model was relaxed for 10 ns at 300 K within the NPT (constant atom number, constant pressure, and constant temperature) ensemble under periodic boundary conditions.

In addition, based on the modified structural model of the as-prepared sample, a vacancy-centered cluster was created by removing the center atom. Subsequently, this model was simulated by another MD, using the same EAM potential and was within the NPT. The details were the same as those described above.
